# Intravitreal Bevacizumab (Avastin) for Diabetic Retinopathy: The 2010 GLADAOF Lecture

**DOI:** 10.1155/2011/584238

**Published:** 2011-03-30

**Authors:** J. Fernando Arevalo, Juan G. Sanchez, Andres F. Lasave, Lihteh Wu, Mauricio Maia, Sergio Bonafonte, Miguel Brito, Arturo A. Alezzandrini, Natalia Restrepo, Maria H. Berrocal, Mario Saravia, Michel Eid Farah, Jans Fromow-Guerra, Virgilio Morales-Canton

**Affiliations:** ^1^Retina and Vitreous Service, Caracas Clinical Opthalmology Center, Caracas 1010, Venezuela; ^2^Retina and Vitreous Service, Instituto Nacional de Investigación en Oftalmologica (INIO), Medellín, Colombia; ^3^Retina and Vitreous Service, Instituto de Cirugia Ocular, San José, Costa Rica; ^4^Departamento de Oftalmologica, Instituto da Visão, Universidad Federal de São Paulo, 04021-001 São Paulo, SP, Brazil; ^5^Retina and Vitreous Service, Centro de Oftalmología Bonafonte, Barcelona, Spain; ^6^Retina and Vitreous Service, Instituto Docente de Especialidades Oftalmológicas (IDEO), Maracaibo, Venezuela; ^7^OFTALMOS, Facultad de Medicina, Universidad de Buenos Aires, Buenos Aires, Argentina; ^8^Department of Ophthalmology, University of Puerto Rico, San Juan, Puerto Rico; ^9^Department of Ophthalmology, Hospital Universitario Austral, Buenos Aires, Argentina; ^10^Hospital Dr. Luis Sanchez Bulnes, Asociación Para Evitar la Ceguera en Mexico 04030, Mexico City, Mexico

## Abstract

This paper demonstrates multiple benefits of intravitreal bevacizumab (IVB) on diabetic retinopathy (DR) including diabetic macular edema (DME) and proliferative diabetic retinopathy (PDR) at 24 months of followup. This is a retrospective multicenter interventional comparative case series of intravitreal injections of 1.25 or 2.5 mg of bevacizumab for DME, PDR without tractional retinal detachment (TRD), and patients who experienced the development or progression of TRD after an intravitreal injection of 1.25 or 2.5 mg of bevacizumab before vitrectomy for the management of PDR. The results indicate that IVB injections may have a beneficial effect on macular thickness and visual acuity (VA) in diffuse DME. Therefore, in the future this new therapy could complement focal/grid laser photocoagulation in DME. In PDR, this new option could be an adjuvant agent to panretina photocoagulation so that more selective therapy may be applied. Finally, TRD in PDR may occur or progress after IVB used as an adjuvant to vitrectomy. Surgery should be performed 4 days after IVB. Most patients had poorly controlled diabetes mellitus associated with elevated HbA1c, insulin administration, PDR refractory to panretinal photocoagulation, and longer time between IVB and vitrectomy.

## 1. Introduction

Diabetic retinopathy remains the major threat to sight in the working age population in the developed world. Furthermore, it is increasing as a major cause of blindness in other parts of the world especially in developing countries [1]. Diabetic macular edema (DME) is a manifestation of diabetic retinopathy that produces loss of central vision. Macular edema within 1 disk diameter of the fovea is present in 9% of the diabetic population [2]. Although visual loss secondary to proliferative changes is more common in patients with type 1 diabetes, visual loss in patients with type 2 diabetes is more commonly due to macular edema [3]. Proliferative diabetic retinopathy (PDR) is a major cause of visual loss in diabetic patients. In PDR, the growth of new vessels from the retina or optic nerve, is thought to occur as a result of vascular endothelial growth factor (VEGF) release into the vitreous cavity as a response to ischemia [4–6]. 

Vascular endothelial growth factor has been shown to be an endothelial cell-specific mitogen and an angiogenic inducer in a variety of in vitro and in vivo models [7]. VEGF, also known as vascular permeability factor, has been demonstrated to increase retinal vessel permeability by increasing the phosphorylation of tight junction proteins. Also, hypoxia has been shown to be a major inducer of VEGF gene transcription [7]. Recent work has found elevated levels of VEGF in ocular fluids of patients with PDR [4–6, 8]. Furthermore, injection of VEGF into normal primate eyes induces the same pathological processes seen in diabetic retinopathy, including microaneurysm formation and increased vascular permeability [9, 10]. Because VEGF has been shown to play a major role in macular edema and retinal neovascularization (RN) [4, 5] although other factors may be involved as well [9, 11] anti-VEGF treatments have been hypothesized as an alternative adjunctive treatment for DME [12, 13] and RN [14–16]. 

Bevacizumab (Avastin, Genentech Inc., San Francisco, CA) is a complete full length humanized antibody that binds to all subtypes of VEGF and is successfully used in tumor therapy as a systemic drug [17]. Recent studies have demonstrated the usefulness of an intravitreal injection of bevacizumab in the reduction of macular edema secondary to central retinal vein occlusion, vascular permeability, and fibrovascular proliferation in RN secondary to PDR, and choroidal neovascularization (CNV) secondary to age-related macular degeneration (AMD) [13, 15, 16, 18–22]. The amount of human retinal penetration for a complete full-length anti-VEGF antibody is not known at present. However, full thickness retinal penetration of intravitreal bevacizumab was observed in an animal model [23, 24]. Additionally, intravitreal bevacizumab does not appear to be toxic to the albino rabbit retina at a concentration of up to 2.5 mg [25]. 

In an open label uncontrolled clinical study of 4303 injections in human eyes with 1.25 mg or 2.5 mg IVT bevacizumab, our group [26] found systemic adverse events in 18 patients (1.5%). These included 7 (0.59%) cases of an acute elevation of systemic blood pressure, 6 (0.5%), cerebrovascular accident; 5 (0.4%), myocardial infarction; 2 (0.17%), iliac artery aneurysms; and 2 (0.17%), toe amputations as well as 5 (0.4%) deaths. Ocular complications included 7 (0.16%) cases of bacterial endophthalmitis, 7 (0.16%) cases of tractional retinal detachment, 4 (0.09%) cases of uveitis, and a case (0.02%) each of rhegmatogenous retinal detachment and vitreous hemorrhage. Bevacizumab appears to be safe and well tolerated during the first 12 months.

Recently, it has been reported that intravitreal injection of bevacizumab may be also useful for early vitreous hemorrhage in PDR in order to decrease the risk of new hemorrhages while clearing occurs and to minimize the indications of vitrectomy [15]. In addition, Chen and Park [27] and Avery et al. [21] have suggested that preoperative intravitreal bevacizumab might be helpful to facilitate vitrectomy in severe PDR cases. In such cases, the preoperative use of bevacizumab might reduce the risk of intraoperative bleeding facilitating the removal of fibrovascular membranes particularly when preoperative PRP cannot be placed. We have previously reported 11 eyes (patients) out of 211 intravitreal injections with development or progression of tractional retinal detachment (TRD) with decrease best-corrected visual acuity (BCVA) after intravitreal bevacizumab prior to vitrectomy for the management of PDR for an incidence of 5.2% [28]. 

 The purpose of this paper is to describe the anatomic and functional outcomes, and the effectiveness of intravitreal bevacizumab (IVB) in patients with diabetic retinopathy at 24 months of followup.

## 2. Primary Intravitreal Bevacizumab (Avastin) for Diffuse Diabetic Macular Edema

Diabetic macular edema is a manifestation of diabetic retinopathy that produces loss of central vision. DME is caused by excessive vascular permeability, resulting in the leakage of fluid and plasma constituents, such as lipoproteins into the retina, leading to thickening of the retina.

Although the Early Treatment Diabetic Retinopathy Study (ETDRS) [29] demonstrated that immediate focal photocoagulation reduced moderate visual loss by 50% (from 24% to 12%, three years after initiation of treatment), 12% of treated eyes still lost 15 or more ETDRS letters at the 3-year follow-up interval. Approximately 40% of treated eyes that had retinal thickening involving the center of the macula at baseline still had thickening involving the center at 12 months, as did 25% of treated eyes at 36 months. Furthermore, only 3% of laser-treated eyes experienced a gain of 3 or more lines of vision. This suggests that a distinct subgroup of eyes exists with DME resistant to conventional laser photocoagulation. Other studies have reported a poor prognosis despite laser photocoagulation in eyes with diffuse DME [29–31]. 

Given that most eyes with DME that are treated with laser photocoagulation do not have an improvement in visual acuity, there has been an interest in other treatment modalities such as pharmacologic therapy with oral protein kinase C inhibitors and the use of intravitreal corticosteroids [32, 33]. The use of antibodies targeted at VEGF, is another treatment modality that has generated considerable interest, and is currently being investigated.

It was recently demonstrated that retinal hypoxia plays a role in DME [34] and VEGF, which is upregulated by hypoxia, is likely to contribute to the excessive vascular permeability that results in macular edema in people with diabetes. Several studies have demonstrated not only a correlation of VEGF levels with the severity of diabetic retinopathy, but also a reduction in levels after successful laser treatment of PDR [9, 11]. Thus a rational approach to treating macular edema in these patients would include the use of anti-VEGF agents [14, 15]. Chun et al. reported that ranibizumab (Lucentis, Genentech Inc., San Francisco, CA) therapy has the potential to maintain or improve BCVA and reduce retinal thickness in patients with DME [35]. In addition, intravitreal injections of the aptamer pegaptanib sodium (Macugen; OSI Eyetech Pharmaceuticals, Melville, NY) in patients with DME has been shown to improve visual acuity (VA) and retinal thickening [12]. Cunningham and coworkers reported gains in visual acuity of 10 letters in 34% and 15 letters in 18% of patients with DME following an intravitreal pegaptanib sodium injection in a randomized, double-masked, multicenter trial with a followup of 36 months.

We have recently reported the 24-month anatomic and BCVA response after primary intravitreal bevacizumab (IVB) in patients with DME [13]. We conducted a multicenter retrospective study of eyes with diffuse diabetic macular edema (DDME) treated with off-label IVB between September 2005 and July 2006 at eleven institutions in Venezuela, Colombia, Costa Rica, Brazil, Argentina, Spain, Peru, and Mexico. We reviewed the clinical records of 115 consecutive patients (139 eyes) with DDME treated with at least one intravitreal injection of 1.25 mg or 2.5 mg of bevacizumab. The dose of 1.25 mg or a dose of 2.5 mg to be used to treat a patient was determined at the discretion of the treating physician. All patients with a minimum followup of 24 months. Our patients had a mean age of 59.4 ± 11.1 years, and 51.3% were male. In that study, patients had a glycosylated hemoglobin (HbA1c) mean of 9.1 ± 1.86%. Regarding the severity of diabetic retinopathy (DR), 17 (12.2%) eyes had mild DR, 25 (18%) eyes had moderate DR, 39 (28.1%) eyes had severe DR, and 58 (41.7%) eyes had PDR. All these 58 cases with PDR had had prior scatter panretinal photocoagulation (PRP) at least 6 months before undergoing IVB. All eyes had DDME diagnosed by biomicroscopic slit-lamp examination, fluorescein angiography (FA), and optical coherence tomography (OCT) (Stratus OCT, Carl Zeiss, Dublin, CA) at baseline. 

Exclusion criteria included patients (eyes) with DME previously treated with laser photocoagulation or intravitreal triamcinolone, macular ischemia, and the presence of an epiretinal membrane or vitreomacular traction syndrome. Patients received reinjections whenever there was a recurrence of DDME. Recurrence was defined as a decrease of BCVA associated with an increase of intraretinal fluid due to macular edema on OCT (≥50 *μ*m in central macular thickness) and/or FA, after complete or partial resolution in previous followup visits.

**Figure 1:**
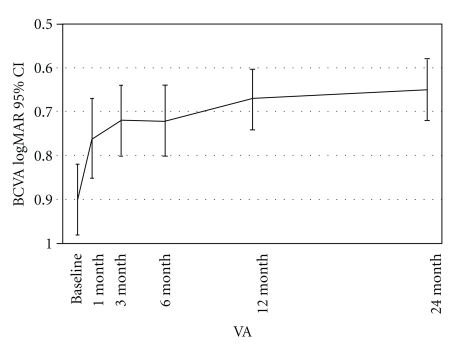
Changes in best-corrected visual acuity (BCVA) after intravitreal bevacizumab. BCVA improved at 1 month from 0.90 to 0.76 (logarithm of the minimum angle of resolution), a difference that was statistically significant (*P* < .001), this level of BCVA was maintained throughout 3-, 6-, 12-, and 24-months. CI: confidence interval (Reprinted with permission from [13]).

Within one month after the initial bevacizumab injection, improvements in BCVA and central macular thickness (CMT) measurements were observed and these significant changes continued throughout the 24-month followup. At one month BCVA improved from log MAR = 0.90 to 0.76, a difference that was statistically significant (*P* < .001) (*P* = .0001). This improvement in BCVA was maintained throughout the 3-, 6-, 12-, and 24-month followup, (Figure 1). In addition, the mean BCVA at 24 months was 20/100, log MAR = 0.70 (*P* < .001) a statistically significant difference from baseline BCVA. Twenty-four month BCVA analysis by subgroups demonstrated that 62 (44.6%) eyes remained stable, 72 (51.8%) eyes improved two or more ETDRS lines of BCVA, and 5 (3.6%) eyes decreased two or more ETDRS lines of BCVA.

**Figure 2:**
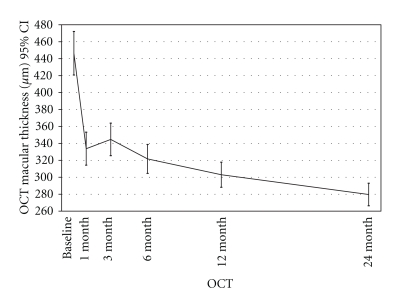
Changes in macular thickness with optical coherence tomography (OCT) during followup after intravitreal bevacizumab. The foveal thickness improved after 1 month, mean 1-mm central macular thickness (CMT) measurement decreased from 446.4 *μ*m ± 154.4 *μ*m to 333.75 *μ*m ± 117 *μ*m (*P* < .001), and this overall improvement continued throughout the 24-month followup. At 3-, 6- 12- and 24-month followup were 344.7 ± 115.3 *μ*m, 321.7 ± 102.7 *μ*m, 303 ± 89.1 *μ*m and, 279.7 ± 80 *μ*m, respectively, which were significantly lower than at 1-month followup (*P* < .001). CI: confidence interval (Reprinted with permission from Arevalo JF, Sanchez JG, Wu L, Maia M, Alezzandrini AA, Brito M, Bonafonte S, Lujan S, Diaz-Llopis M, Restrepo N, Rodríguez FJ, Udaondo-Mirete P; Pan-American Collaborative Retina Study Group. Primary intravitreal bevacizumab for diffuse diabetic macular edema the Pan-American Collaborative Retina Study Group at 24 months. Ophthalmology 2009; 116:1488-97, 1497.e1. Epub 2009 Jul 9).

**Figure 3:**
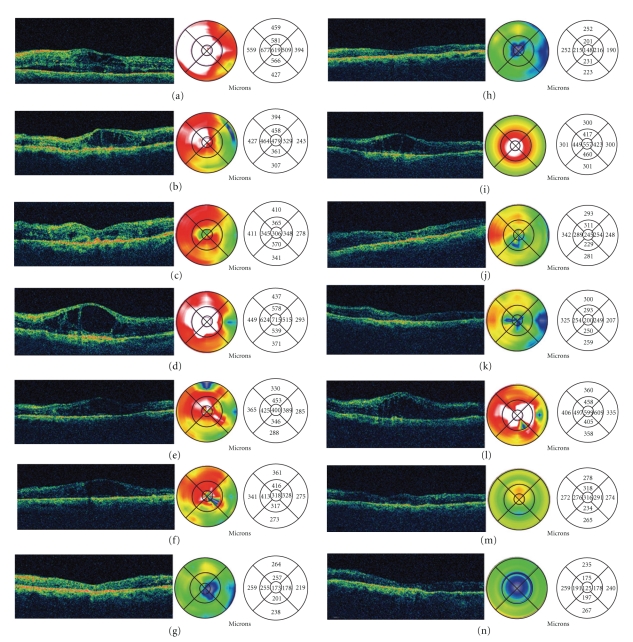
Sequential optical coherence tomography (OCT) of a 69-year-old diabetic woman with a 6-months history of lost of vision to counting fingers (CF) in her left eye that had developed diabetic macular edema (DME).

Optical coherence tomography results were available for all 139 eyes at 1-, 3-, 6-, 12-, and 24-month followups. At one month, the mean 1-mm CMT measurements decreased from 446.4 *μ*m ± 154.4 *μ*m to 333.75 *μ*m ± 117 *μ*m (*P* < .001), and this overall improvement continued throughout the 24-month followup, (Figures 2 and 3). At 3-, 6-, 12-, and 24-month follow-ups, mean CMT were 344.7 *μ*m ± 115.3 *μ*m, 321.7 *μ*m ± 102.7 *μ*m, 303 *μ*m ± 89.1 *μ*m, and 279.7 ± 80, respectively, which were significantly different from baseline (*P* < .001). 

In Figure 3(a) a horizontal OCT scan obtained through the fovea revealed loss of the normal foveal contour, diffuse macular thickening, areas of low intraretinal reflectivity consistent with intraretinal cysts, and subretinal fluid (SRF). The retinal map analysis revealed a foveal thickness of 619 *μ*m. The patient underwent an intravitreal injection of bevacizumab at a dose of 2.5 mg in this eye. In Figure 3(b) OCT reveals partial resolution of intraretinal macular edema and complete reabsorption of SRF at one month after bevacizumab injection. The retinal map analysis indicates a central foveal thickness of 479 *μ*m. Visual acuity (VA) improved to 20/400. In Figure 3(c) 3 months after the injection, the OCT scan shows improvement in foveal thickness (306 *μ*m). VA improved to 20/200. In Figure 3(d) four months after the first injection, her VA diminished to CF, and OCT scan showed the reappearance of macular edema associated to the increase of intraretinal cysts. Central foveal thickness increased to 715 *μ*m. She received a second injection of intravitreal bevacizumab at a dose of 2.5 mg at this point. In Figures 3(e) to 3(g), at month six she received a third injection of intravitreal bevacizumab at dose de 2.5 mg. OCT scans at 5, 6, and 9 months showed a progressive resolution in macular edema and intraretinal cysts, which were confirmed with decrease of central foveal thickness (400 *μ*m, 318 *μ*m, and 173 *μ*m, resp.). VA also improves progressively (20/200, 20/200, and 20/125, resp.). In Figure 3(h) twelve months after the first injection, the OCT scan showed resolution of DME, with complete reabsorption of SRF and restoration of foveal anatomy. Foveal thickness decreased to 148 *μ*m, and visual acuity was 20/125. In Figure 3(i) sixteen months after the first injection, her VA diminished to 20/400, and the OCT scan showed a reappearance of macular edema associated to increased of intraretinal cysts. Central foveal thickness increased to 557 *μ*m. She received a fourth injection of intravitreal bevacizumab at dose de 2.5 mg. In Figure 3(j) OCT scan at 17 months showed a resolution in macular edema and intraretinal cysts. Central foveal thickness decreased to 245 *μ*m and VA was 20/160. In Figure 3(k) 18 months after the first injection (2 months after the previous injection), the OCT scan shows improvement in foveal thickness (200 *μ*m). VA improved to 20/125. In Figure 3(l) nineteen months after the first injection, his visual acuity diminished to 20/400, and the OCT scan showed the reappearance of macular edema. The retinal map analysis indicates a central foveal thickness of 599 *μ*m. She received a fifth injection of intravitreal bevacizumab at a dose of 2.5 mg at this point. In Figure 3(m) OCT scan at twenty months showed resolution in macular edema and intraretinal cysts. Central foveal thickness decreased to 316 *μ*m. VA improved to 20/200. In Figure 3(n) twenty-four months after the first injection, OCT showed a marked resolution in macular edema and restoration of foveal anatomy. Central foveal thickness was 125 *μ*m, and VA improved to 20/160 (Reprinted with permission from [13]).

We wished to compare the response to treatment between patients with PDR and previous PRP to those with nonproliferative diabetic retinopathy and DDME to see if there was any difference. However, when we ran the repeated measures ANOVA to compare mean values to statistically analyze mean retinal thickness and logMAR VA adjusting for the grade of diabetic retinopathy as a covariate, we found no statistical significance (*P* = .511 for BCVA and *P* = .483 for CMT).

All eyes received an intravitreal injection at the initial visit; however, recurrences were retreated at the discretion of the treating physician. There were a total of 807 IVB injections performed. The mean number of IVB injections per eye was 5.8 (range: 1 to 15 injections) at a mean interval of 12.2 ± 10.4 weeks. Seventy-four (53.2%) cases were treated with an intravitreal injection of 1.25 mg of bevacizumab and sixty-five (46.8%) cases with at a dose of 2.5 mg of IVB. 

Adverse events included transient high blood pressure in one (0.9%) patient, cerebrovascular accident in one (0.9%) patient, heart attack in one (0.9%) patient, transient increased intraocular pressure in seven (5%) eyes, cataract in five (3.6%) eyes, and tractional retinal detachment in one (0.7%) eye. There were no episodes of inflammation or severe decrease of vision immediately after an injection. 

Our results indicate that primary IVB at doses of 1.25 mg or 2.5 mg seems to provide stability and improvement in BCVA, OCT, and FA in DDME at 24 months. We identified no difference in outcomes between IVB at doses of 1.25 mg or 2.5 mg. Therefore, doses lower than 2.5 mg should be preferred. These results indicate that IVB injections may have a beneficial effect on macular thickness and BCVA in DDME.

## 3. Intravitreal Bevacizumab (Avastin) for Proliferative Diabetic Retinopathy

Panretinal photocoagulation (PRP) has been the mainstay for the treatment of PDR, and its suppressive effect on RN has been well documented [36–39]. However, substantial regression of new vessels may take weeks after completion of PRP, and in up to one third of cases, new vessels continue to grow despite initial PRP [37, 39]. In these cases, vitreous hemorrhage may induce visual loss and prevent complete laser. Moreover, macular edema may increase after PRP and cause transient or persistent visual loss [40, 41]. 

Neovascularization on and around the optic disc (NVD) and vitreous hemorrhage were found to be more frequently associated with severe visual loss despite PRP in the Diabetic Retinopathy Study (DRS) and ETDRS [42, 43]. Long intervals between PRP sessions and the variable amount of time required for a favorable response may increase the incidence of complications due to the progression of PDR [36, 42]. In fact, a single episode of PRP or shorter intervals between PRP episodes, although desirable in severe PDR and when the patient must travel long distances for treatment, are often associated with acute visual disturbances due to exudative choroidal detachment, retinal detachment, and macular edema [37, 44–46]. 

Although RN actually may be due to more than one cytokine, VEGF is an important, if not the most important cytokine involved [47]. Activation of the VEGF-receptor pathway triggers a network of signaling processes that promotes endothelial cell growth, migration, survival from preexisting vessels, differentiation, and mobilization of endothelial progenitor cells from the bone marrow into the peripheral circulation [17, 48, 49]. Furthermore, VEGF increases vessel permeability leading to deposition of proteins in the interstitium that facilitate the process of angiogenesis [50]. There are several reports published on the intravitreal administration of anti-VEGF compounds for RN in diabetic retinopathy [14, 21]. In addition, there are several case reports on the use of intravitreal bevacizumab in RN in diabetic retinopathy demonstrating regression of RN in PDR [22, 27, 51–53]. 

We conducted a retrospective study in 43 eyes of 39 patients with PDR that had retinal neovascularization (RN), who were treated with off-label intravitreal bevacizumab between September 2005 and July 2007 at 7 institutions in Venezuela, Costa Rica, Brazil, Argentina, Spain, and Peru. Patients were followed for 24 months. Our patients had a mean age of 54 ± 12.5 years old (range from 28 to 79 years), and twenty-three (59%) patients were male. Eleven (28.2%) diabetic patients were insulin dependent. The mean duration of diabetes was 17.2 years (range from 1 to 33 years). Twenty-four (55.8%) eyes were treated with an intravitreal injection of 1.25 mg and 19 (44.2%) eyes were treated with 2.5 mg of bevacizumab. Of the total of 43 eyes, 31 (72.1%) eyes had been previously treated with scatter photocoagulation at least 6 months before IVB, no eyes had prior focal/grid laser photocoagulation, and no eyes had a previous intravitreal triamcinolone injection. All eyes had clinical significant macular edema (CSME) at biomicroscopic noncontact fundus examination with a 66- or a 78-diopter lens. 

The mean baseline BCVA was log MAR = 0.94 ± 0.38 ETDRS (20/176) and the mean 24-month BCVA was log MAR = 0.67 ± 0.39 ETDRS (20/94) (*P* < .0005). Final BCVA analysis by subgroups demonstrated that 35 (81.4%) eyes remained stable, 5 (11.6%) eyes improved two or more ETDRS lines of BCVA, and 3 (7%) eyes decreased two or more ETDRS lines of BCVA. 

Optical coherence tomography results were available for all 43 eyes at 1-, 3-, 6-, 12-, and 24-month follow-up time points. At 1 month, the mean 1-mm central macular thickness (CMT) measurements decreased from 430.9 *μ*m ± 169.5 *μ*m to 311.51 *μ*m ± 116 *μ*m (*P* < .05) (*P* = .01), and this overall improvement continued throughout the 24-month follow-up. At 3-, 6-, 12-, and 24-month follow-ups, mean CMT were 319.6 *μ*m ± 95.7 *μ*m, 297.1 *μ*m ± 102.5 *μ*m, 297.5 *μ*m ± 91.2 *μ*m and 270.3 ± 75.9 respectively, which were significantly different from baseline (*P* = .0001). 

**Figure 4:**
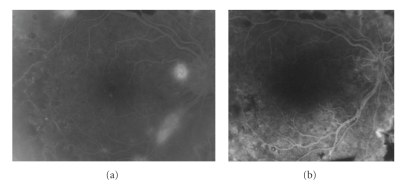
A 53-year-old man had a 2-month history of visual loss to 20/60 in his right eye. We had performed panretinal photocoagulation (PRP) in his right eye 2 years previously. Fundus examination revealed a mild vitreous hemorrhage. (a) Fluorescein leakage from neovascularization of the disc (NVD) at baseline between retinal vessels crossing the optic disc was demonstrated. In addition, fluorescein angiography (FA) showed magnification of retinal neovascularization elsewhere (NVE) in the superonasal retina. (b) At week 1 after intravitreal bevacizumab, total resolution of leakage from NVD and NVE are shown. His visual acuity returned to 20/32 one month later. He needed a reinjection at months 6, 14, and 24 of followup. A PRP was performed at 24 months.

 Of the total of 43 eyes, 17 (39.5%) eyes treated showed total regression of RN on fundus examination with absence of fluorescein leakage, (Figure 4), 15 (34.9%) eyes demonstrated partial regression of RN on fundus examination and FA, and 11 (25.6) eyes showed no regression of RN (Table 1).

When divided by type of RN, of the total of 43 eyes, 15 (34.9%) eyes showed total regression of NVE (neovascularization elsewhere) on fundus examination with absence of fluorescein leakage, (Figure 4), 10 (23.3%) eyes demonstrated partial regression of NVE on fundus examination and FA, and twelve (27.9%) eyes showed no regression of NVE. Thirteen (30.2%) eyes showed total regression of NVD on fundus examination with absence of fluorescein leakage, 14 (32.5%) eyes demonstrated partial regression of NVD on fundus examination and FA, and sixteen (37.2%) eyes showed no regression of NVD.

When divided by IVB dose utilized, we observed that 9 (37.5%) eyes treated with 1.25 mg dose showed no regression of NVE and 13 (54.2%) eyes showed no regression of NVD at the end of followup, while only 3 (15.8%) eyes treated with 2.5 mg dose showed no regression of NVE, and 3 (15.8%) eyes showed no regression of NVD at 24 months of followup. These differences were statistically significant (*P* = .02) and (*P* = .0001), respectively. 

The mean number of IVB injections per eye was 4.1 ± 2.1 (range: 1 to 8 injections) at a mean interval of 14.8 ± 10.4 wks. Twenty-one eyes (47.7%) needed a second injection due to recurrence of RN at a mean of 12.4 weeks (range from 4 to 34 weeks), and seven eyes (15.9%) needed a third injection due to recurrence of neovascularization at a mean of 17.3 weeks (range from 11 to 22 weeks). 

Three patients without previous PRP (“naive”) and with vitreous hemorrhage have avoided vitreoretinal surgery. There were no episodes of inflammation or severe loss of vision immediately after an injection. Regarding adverse events at 24 months, two thromboembolic events were reported, a cerebrovascular accident in 1 patient (2.6%) and a myocardial infarction in 1 patient (2.6%). One TRD was reported (2.3%), and one eye (2.3%) developed a vitreous hemorrhage. 

To determine the effect of an intravitreal injection of bevacizumab on actively growing new vessels, we chose the variation in vitreous leakage from RN as our primary outcome. The detection of NVD and NVE on FA allowed the use of a systematic anatomical approach to monitor the area of leaking new vessels over time. Finally, to determine the effect of an intravitreal injection of bevacizumab on macular edema, we measured the variation on retinal thickness with OCT. 

Our studies have demonstrated that intravitreal bevacizumab resulted in marked regression of RN on fundus examination and FA in patients with PDR and previous PRP, specially in the first six months [16]. Furthermore, a rapid resolution of vitreous hemorrhage in 3 naive eyes was seen. In addition, IVB demonstrated a similar beneficial response on macular thickness in eyes with PDR, and probably bevacizumab prevents exacerbation of macular edema in patients with concomitant CSME and PDR. 

There was a tendency towards a decrease in response to IVB overtime with 60.5% of eyes at 24 months requiring additional PRP or vitrectomy, which raises the question of tachyphylaxis. However, further analysis of our data seems to rule out this possibility.

Intravitreal bevacizumab seems to be a useful treatment for PDR, minimizing the risk for exudative complications, progression of retinal neovascularization, vitreous hemorrhage, and decreased vision caused by macular edema. Intravitreal bevacizumab may potentially be used as an adjuvant agent to PRP for PDR.

## 4. Tractional Retinal Detachment following Intravitreal Bevacizumab (Avastin) in Patients with Severe Proliferative Diabetic Retinopathy

In our updated retrospective review, we have identified 25 eyes (patients) out of 698 IVT injections that developed or had progression of TRD with decrease of BCVA after intravitreal bevacizumab prior to vitrectomy for the management of PDR for an incidence of 3.5%.

 All patients had had a PRP at least 2 months before intravitreal bevacizumab. All eyes had PDR refractory to PRP. The mean age of the study group was 61 ± 8.5 years (range from 24 to 76 years), and 52% were female. Eighteen (72%) patients had DM type 2 with a mean of 14 ± 5.9 years from diagnosis (range: 1–25 years) and seven (28%) patients had DM type 1 with a mean of 16.3 ± 8 years from diagnosis (range: 7 to 30 years). In the current study, eleven (44%) patients used insulin administration as sole treatment for glycemic control, seven (28%) diabetic patients controlled glycemic levels with oral therapy, and the remainder seven (28%) patients used combination therapy with insulin and oral hypoglycemic agents for glycemic control. However, all patients had uncontrolled diabetes associated with elevated glycosylated hemoglobin (HbA1c mean = 9.2%). Eleven (44%) eyes had local TRD on indirect ophthalmoscopy, ultrasound, or biomicroscopic noncontact fundus examination with a 66- or a 78-diopter lens before intravitreal bevacizumab.

**Figure 5:**
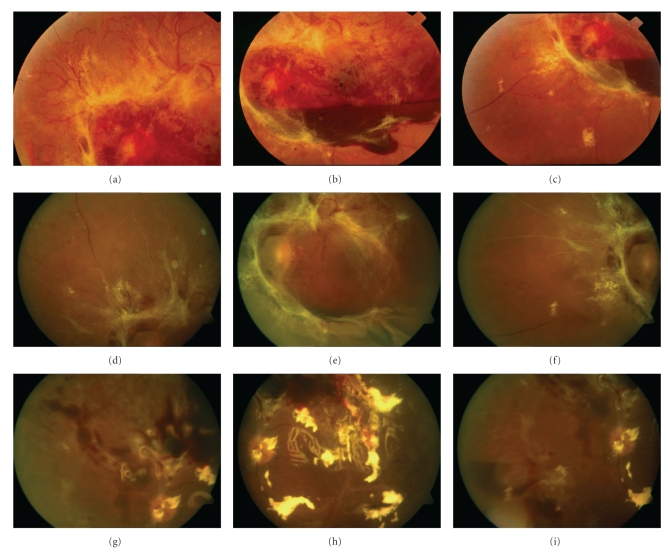
(a)–(c) Color photographs before intravitreal bevacizumab. Severe proliferative diabetic retinopathy with abundant fibrovascular tissue and subhyaloid hemorrhage. The retina is attached and best-corrected visual acuity (BCVA) is 20/80. (d)–(f) Color photographs 10 days after 2.5 mg of intravitreal bevacizumab demonstrating dense fibrous tissue contraction, and tractional retinal detachment with macular involvement. BCVA is hand motions at 2 meters. (g)–(i) Same eye, eight days after vitrectomy. The retina is reattached and best-corrected visual acuity (BCVA) is 20/120 with silicone oil tamponade.

Twenty-five (3.2%) eyes (patients) out of 698 IVT injections developed or had progression of TRD, (Figure 5). Nineteen (73%) eyes had received a dose of 1.25 mg (out of 626 injections: 3%), and 6 (27%) eyes had received a dose of 2.5 mg (out of 72 injections: 8.3%). Time from injection to TRD had a mean of 11 ± 7.5 days (range from 5 to 32 days) and time from injection to vitrectomy had a mean of 18.8 ± 11.5 days (range from 5 to 37 days).

 The mean baseline (before intravitreal bevacizumab) BCVA was log MAR = 1.4 ± 0.7 (range from 0.2 to 2.9) (mean ETDRS equivalent: 20/400; range: 20/32 to NLP). At TRD development or progression, the mean BCVA was log MAR = 1.9 ± 0.6 (range: 0.3 to 2.9) (mean ETDRS equivalent: CF; range: 20/40 to NLP), this difference was statistically significant compared to baseline BCVA (*P* ≤ .0001). One patient developed a retinal break as a result of the increased traction, and a combined total tractional-rhegmatogenous retinal detachment was apparent 3 weeks after intravitreal bevacizumab.

Twenty-two eyes underwent vitrectomy, two patients refused or were unable to undergo surgery, and in one patient surgery was not recommended. Vitrectomy was performed in a mean of 18.8 ± 11.5 days (range from 5 to 37 days) after intravitreal injection of bevacizumab. Tractional retinal detachments were managed with vitrectomy, membranectomy, photocoagulation, and extended intraocular tamponade with gas in all patients that underwent surgery. Final mean BCVA after surgery was log MAR = 1.4 (range: 0.2 to 2.9) (mean ETDRS equivalent: 20/400; range: 20/32 to NLP), this difference was statistically significant compared to TRD BCVA (*P* = .012). Subgroup analysis of final BCVA after vitrectomy demonstrated that 12 (54.5%) out of 22 eyes improved two or more ETDRS lines of BCVA when compared to TRD BCVA. However, when compared to baseline BCVA, final BCVA after vitrectomy demonstrated that 8 (36.4%) eyes improved, 5 (22.7%) eyes remained stable, and 9 (40.9%) eyes lost two or more ETDRS lines of BCVA.

It could be argued that TRD may develop soon after extensive PRP in diabetes. In addition, all our patients were refractory to extensive PRP. However, all patients had had a PRP at least 2 months before intravitreal bevacizumab. The short time between the injection and TRD suggest a cause-effect relationship. It also suggests that in cases at risk for progression of TRD that might involve the central macular region, timely surgery should be anticipated following intravitreal bevacizumab. All patients had TRDs developed or progressed 5 days or more after the injection. Therefore, surgery should be performed 4 days after IVB. In addition, most of these patients had poorly controlled diabetes mellitus associated with elevated HbA1c, insulin administration, PDR refractory to panretinal photocoagulation, and longer time between intravitreal bevacizumab and vitrectomy.

## 5. Risk Factors for the Development or Progression of Tractional Retinal Detachment following Intravitreal Bevacizumab (Avastin) in Patients with Severe Proliferative Diabetic Retinopathy

Clinical parameters of patients previously identified as potential risk factors for TRD were obtained and analyzed and compared to the clinical characteristics of those patients from the same cohort that did not develope a TRD after IVB for PDR. We analyzed the presence or absence of 20 potential risk factors for all patients with a TRD and compared them to patients that did not develop a TRD. These potential risk factors included systemic and surgical background, age, time from diabetes mellitus (DM) diagnosis, glycemic control, cholesterol levels, triglycerides levels, hemoglobin A1c (HbA1C), dose of bevacizumab, and time from injection to vitrectomy. 

Twenty-Five eyes (patients) (3.5%) out of 698 intravitreal injections developed or had progression to a TRD after intravitreal bevacizumab. Patients with TDR were followed for an average of 48.9 weeks (range: 0 to 190 weeks), and had a mean age of 54.4 ± 11.5 years. Twelve were male (47.8%) and thirteen were females (52.2%). Thirteen patients (52%) were Hispanic, 6 (24%) were Caucasian, and 6 (24%) were African-American. Seventeen (68%) had diabetes type 2 and 8 (32%) had diabetes type 1. In the current study, twenty (80%) patients with TRD had a previous PRP and had an average HbA1c before treatment of 9.85 ± 2.6%. Seventeen (68%) had less than 15 years diagnosed with DM and 8 (32%) had more than 15 years with a diagnosis of DM. 

Patients without TRD were followed for an average of 33.1 weeks (range: 0 to 170 weeks) and had a mean age of 53.94 ± 12.61 years. Four hundred and one were male (59.6%) and 272 were female (40.4%). Four hundred and fifty (66.9%) were Hispanic, 122 (18.1%) were Caucasian, and 101 (15%) were African-American. One hundred ninety patients (28.4%) had diabetes type 1 and 482 (71.6%) had diabetes type 2. Four hundred seventy-two (70.15%) patients without TRD had a previous PRP and had an average HbA1c before treatment of 9.08 ± 2.11%. In addition, 242 (36.6%) had less than 15 years of diagnosis with DM and 426 (63.3%) had more than 15 years with a diagnosis of DM.

Of these 698 intravitreal injections, 626 applications were with 1.25 mg of bevacizumab (of which 19 patients had TRD, 3%) and 72 injections with 2.5 mg of bevacizumab (of which 6 patients had TRD, 8.3%). No systemic adverse events such as thromboembolic events (cerebrovascular accidents, transient ischemic attacks, myocardial infarctions, or peripheral vascular disease) were reported. All eyes had TRD diagnosed by indirect ophthalmoscopy, fundus biomicroscopy, FA, and OCT at baseline. 

Risk factors for TRD after IVB identified in our study included, more than 15 years from the diagnosis of diabetes mellitus (DM) (*P* = .009), (OR = 0.30), (95CI = 0.10–0.83), (RR = 0.35), more than 13 days from injection to vitrectomy (*P* = .0001), (OR = 9.9), (95CI = 3.4–29), (RR = 6.9) and the use of a higher dose (2.5 mg) of bevacizumab (*P* = .022), (OR = 2.7), (95CI = 1.05–7.18), (RR = 2.38). 

We did not find a statistical associations between others risk factors and TRD, such as macular thickness (*P* = .123), neovascular membrane (*P* = .145), or previous use of bevacizumab (*P* = .653). Fibrovascular proliferation was present in most patients with TRD (95% CI = 73.9–99.8). However, no significant correlation was found between TRD and fibrovascular proliferation despite that 94.7% of the patients had this factor present (*P* = .260) (Table 2).

## 6. Conclusions

Diabetic macular edema is a manifestation of diabetic retinopathy that produces loss of central vision, and is the most frequent cause of visual impairment in patients with nonproliferative diabetic retinopathy. However, the breakdown of endothelial tight junctions and loss of the blood retinal barrier that lead to DME can be associated with both nonproliferative diabetic retinopathy and PDR. Although several treatment modalities are currently under investigation, the only demonstrated means to reduce the risk of vision loss from DME are laser photocoagulation, as demonstrated by the ETDRS, [29] intensive glycemic control, as demonstrated by the Diabetes Control and Complications Trial (DCCT) and the United Kingdom Prospective Diabetes Study (UKPDS) and blood pressure control, as demonstrated by the UKPDS [54, 55]. 

The results of our retrospective study demonstrated the efficacy of 1.25 mg or 2.5 mg of intravitreal bevacizumab as primary treatment of DME as 51.8% of eyes showed anatomical as well as functional improvement. In addition, our results suggest a reduced risk of VA loss in eyes with DME treated with intravitreal bevacizumab (97.1% of eyes). All eyes received an intravitreal injection at the initial visit; however, recurrences were retreated at the discretion of the treating physician. There were a total of 807 IVB injections performed. At 24 months, the mean number of IVB injections per eye was 5.8 (range: 1 to 15 injections) at a mean interval of 12.2 ± 10.4 weeks. Seventy-four (53.2%) cases were treated with an intravitreal injection of 1.25 mg of bevacizumab and sixty-five (46.8%) cases with at a dose of 2.5 mg of IVB. The optimum dosing and sequence for intravitreal bevacizumab in DME is still undetermined. We elected to defer reinjections until there was a recurrence. 

Our results indicate that intravitreal bevacizumab injections may have a beneficial effect on macular thickness and VA for DDME. Therefore, in the future this new treatment modality could complement focal/grid laser photocoagulation. Furthermore, focal/grid laser photocoagulation could be used to consolidate the results obtained with one or a series of intravitreal bevacizumab injections and decrease the need for reinjections.

In addition, we demonstrated that intravitreal bevacizumab resulted in marked regression and then stability of RN on fundus examination and FA in patients with PDR and previous PRP. A rapid resolution of vitreous hemorrhage can also be seen. Furthermore, intravitreal bevacizumab demonstrated a similar beneficial response on macular thickness in eyes with PDR, and probably bevacizumab prevents exacerbation of macular edema in patients with concomitant CSME and PDR. Regression of neovascularization and decrease of retinal thickening occurred in some injected eyes as soon as 7 to 15 days after the intravitreal injection of bevacizumab. Twenty-one eyes (47.7%) needed a second injection due to recurrence of neovascularization at a mean of 12.4 weeks, and seven eyes (15.9%) needed a third injection due to recurrence of neovascularization at a mean of 17.3 weeks. We elected to defer reinjection only when there was a recurrence of RN. Interestingly, we noted that RN responded better to IVB at a dose of 2.5 mg than to a dose of 1.25 mg. Nevertheless, the optimum dose and dosing sequence for IVB is still undetermined. Another interesting finding at 24 months is an increase in the number of eyes that did not respond to IVB with complete RN regression as compared to our previously published 6-month data [16]. It is possible that over time the effect of IVB on RN diminishes, and that other means to control RN will be necessary including PRP and vitrectomy. Although one tractional retinal detachment was reported (1 eye; 2.3%) and one (2.3%) eye developed a vitreous hemorrhage in our series, further studies are needed to assess the efficacy and safety of IVB in the management of PDR. 

We have identified 25 eyes (patients) out of 698 IVT injections that developed or had progression of TRD with decrease in BCVA after intravitreal bevacizumab prior to vitrectomy for the management of PDR. The natural course of PDR is characterized by a cycle of proliferation and regression typical of new vessels; proliferation of fibrous tissue accompanying new vessels; formation of adhesions between the fibrovascular proliferations and the posterior vitreous surface; contraction of the posterior vitreous surface and associated proliferation. The development or progression of TRD in PDR following intravitreal bevacizumab in our patients could have happened by natural history or rapid neovascular involution with accelerated fibrosis and posterior hyaloidal contraction as a response to decreased levels of VEGF. In the current study, eleven (44%) patients used insulin administration as sole therapy for glycemic control, seven (28%) diabetic patients controlled glycemic levels with oral therapy and the remainder seven (28%) patients used combination therapy with insuline and oral hypoglycemic agents for glycemic control. They all had uncontrolled diabetes associated with elevated glycosylated hemoglobin (HbA1c mean = 9.2%).

Results of this study suggest that TRD in PDR may occur or progress after intravitreal bevacizumab used as an adjuvant to vitrectomy. However, in the eyes that underwent vitrectomy, we had the impression that there was a reduced risk of intraoperative bleeding facilitating the removal of fibrovascular membranes. A bloodless field allows for better visibility and the surgeon may be less likely to create an iatrogenic retinal break. In addition, the chances of postoperative complications such as rebleeding or fibrinoid syndrome may be decreased. All these advantages may allow us to save more eyes utilizing preoperative intravitreal bevacizumab regardless of increased traction on some severe PDR cases. Moreover, most patients with development or progression of TRD had poorly controlled diabetes mellitus associated with elevated HbA1c, insulin administration, PDR refractory to panretinal photocoagulation, and longer time between intravitreal bevacizumab and vitrectomy. These factors needed to be studied to determine if they are indeed risk factors for the development or progression of TRD after preoperative IVB in PDR.

Finally, we did study the risk factors for the development or progression of TRD after preoperative IVB in PDR. In our retrospective review, we identified 25 eyes (patients) with development or progression of TRD after intravitreal bevacizumab prior to vitrectomy for the management of PDR for an incidence of 3.5%; the risk factors associated with these patients were more than 15 years from the diagnosis of diabetes mellitus, more than 13 days from injection to vitrectomy and the use of a higher dose (2.5 mg) of bevacizumab. Variables such as smoking history, hypertension, myocardial infarction, levels of triglycerides, cholesterol, blood glucose, previous vitreous hemorrhage, and macular thickness were not related to the development or progression of tractional retinal detachment.

Based on our data, we now believe that extreme care must be taken in using a dose of 2.5 mg or more of bevacizumab in patients with PDR. In addition, to have over 15 years with a diagnosis of diabetes can increase the risk of TRD and that careful follow-up evaluation following injection is mandatory. The timing of surgery after the injection is also important, as there are concerns that bevacizumab may cause progression of the TRD. It is important that surgery is performed once the antiangiogenic effect of bevacizumab has fully developed, but before there is further fibrous proliferation; physicians must be prepared to perform the vitrectomy preferably before 13 days after the application of bevacizumab and to perform a vitrectomy immediately on those patients in whom a TRD occurs. The results of this study show that intravitreal bevacizumab therapy is a well-tolerated treatment option for PDR for carefully selected patients, but we can safely assume that the application of bevacizumab may lead to tractional retinal detachment in a proportion of 3.58% (CI 95% = 2.13–5.03) of patients.

## 7. Summary

Intravitreal bevacizumab seems to be a promising treatment for PDR, minimizing the risk for exudative complications, progression of retinal neovascularization, vitreous hemorrhage, and decreased vision caused by macular edema. Intravitreal bevacizumab may potentially be used as an adjuvant agent to PRP for PDR. In addition, primary intravitreal bevacizumab at doses of 1.25 mg or 2.5 mg seems to provide stability and improvement in VA, OCT, and FA in diabetic macular edema. Evaluation in a multicenter, randomized, controlled clinical trial with longer followup is needed to evaluate the safety and efficacy of this new treatment. Tractional retinal detachment in PDR may occur or progress after IVB used as an adjuvant to vitrectomy. Surgery should be performed 4 days after IVB. Based on our multivariate and bivariate analysis, risk factors for TRD after IVB in PDR are time from diagnosis of DM of more than 15 years, time interval from IVB to vitrectomy of more than 13 days, and the use of the higher dose of IVB (2.5 mg).

##  Conflict of Interests

The authors have no financial or proprietary interest in any of the products or techniques mentioned in this paper.

## Figures and Tables

**Table 1:** Regression of RN on fundus examination with absence of fluorescein leakage after IVB at 24 months*.

RN Regression	Unresponsive Neovascularization		Naive Neovascularization		Total eyes
	2.5 mg	1.25 mg	2.5 mg	1.25 mg	
Total	5	7	2	3	17 (39.5%)
Partial	4	7	2	2	15 (34.9%)
No.	3	5	2	1	11 (25.6%)
Total eyes	12	19	6	6	43 (100%)

*RN: Retinal neovascularization, IVB: Intravitreal bavacizumab.

**Table 2:** Risk factors for tractional retinal detachment*.

Risk factor	*P*	CI 95%
More than 15 years from the diagnosis of diabetes mellitus	.009^‡^	0.10–0.83
More than 13 days from injection to vitrectomy	.0001^‡^	3.4–29
Use of a higher dose (2.5 mg) of bevacizumab	.022^‡^	1.05–7.18
Diabetes Type	.833	0.34–2.20
Fibrovascular proliferation	.260	0.38–12.15
History of smoking	.408	0.11–2.41
History of HTA	.534	0.29–1.87
Prior Myocardial Infarction	.10	0.088–2.73
Prior Cerebrovascular Accident	.51	0.33–14.7
Total Cholesterol	.895	0.16–4.53
Triglycerides	.453	0.08–9.12
Hemoglobin levels	.796	0.07–4.40
Macular Thickness of DME	.123	0.33–11.65
Preretinal Hemorrhage	.317	0.33–17.5
Vitreous Hemorrhage	.292	0.24–1.47
Previous vitrectomy	.632	0.49–24.97

*DME: Diabetic Macular Edema; TRD: Tractional Retinal Detachment; HTA: Hypertension.

^‡^Statistically significant.
